# Supplementary material for: Seed Priming with Sorghum Water Extract Improves the Performance of Camelina (Camelina sativa (L.) Crantz.) under Salt Stress
Source: Plants (Basel). 2021 Apr 12;10(4):749. doi: 10.3390/plants10040749 (PMC8069245; doi:10.3390/plants10040749)
Supplement: Supplementary file 1 [file plants-10-00749-s001.pdf]

Table S1: Effects of sorghum water extract (control, hydropriming and 5% SWE), salt stress (control and 10 dS m^{-1} from NaCl) on the germination, morphological and physiological traits of camelina.

Salt stress	Priming amendments	FEP	α-amylase activity	RL	SL	RDW	SDW	Chl a	Chl b
Control	NP	2.65±1.53	0.33±0.19	1.05±0.61	1.53±0.88	0.10±0.06	0.15±0.09	0.10±0.06	0.10±0.06
	HP	3.51±2.03	0.21±0.12	1.00±0.58	1.53±0.88	0.06±0.03	0.10±0.06	0.06±0.03	0.10±0.06
	SWE	5.13±1.45	0.19±0.11	0.90±0.52	1.53±0.88	0.06±0.03	0.10±0.06	0.10±0.06	0.10±0.06
Salt stress	NP	3.06±1.77	0.20±0.12	0.94±0.54	1.00±0.58	0.06±0.03	0.10±0.06	0.10±0.06	0.10±0.06
	HP	3.51±2.03	0.25±0.14	1.00±0.58	1.00±0.58	0.06±0.03	0.10±0.06	0.06±0.03	0.15±0.09
	SWE	2.52±1.45	0.10±0.06	1.00±0.58	1.53±0.88	0.06±0.03	0.10±0.06	0.10±0.06	0.15±0.09
		H_2O_2	MDA	CAT	SOD	POD	Na^+	K^+	
Control	NP	0.35±0.20	0.55±0.32	0.40±0.23	0.55±0.32	0.55±0.32	0.58±0.33	1.00 ±0.58	
	HP	0.88±0.51	0.25±0.14	0.36±0.21	0.42±0.24	0.25±0.14	1.00±0.58	1.00 ±0.58	
	SWE	0.08±0.05	0.37±0.22	0.32±0.19	0.40±0.23	0.37±0.22	1.15±0.67	1.00 ±0.58	
Salt stress	NP	0.19±0.11	0.56±0.32	0.21±0.12	0.35±0.20	0.56±0.32	1.00±0.58	1.00 ±0.58	
	HP	0.34±0.19	0.45±0.26	0.10±0.06	0.32±0.19	0.45±0.26	0.58±0.33	1.00 ±0.58	
	SWE	0.19±0.11	0.39±0.23	0.21±0.12	0.40±0.23	0.39±0.23	1.00±0.58	1.00 ±0.58	

The values are standard deviation (SD) ± Standard error (SE).

FEP, final emergence percentage; RL, root length; SL, shoot length; RDW, root dry weight; SDW, shoot dry weight; Chl a, chlorophyll a; Chl b, chlorophyll b; H_2O_2, hydrogen peroxide; MDA, malondialdehyde; CAT, catalase; SOD, superoxide dismutase; POD, peroxidase; Na^+, sodium ion; K^+, potassium ion; NP, no priming; HP, hydro-priming; SWE, sorghum water extract.
